# Aberrantly expressed long noncoding RNAs in human intervertebral disc degeneration: a microarray related study

**DOI:** 10.1186/s13075-014-0465-5

**Published:** 2014-10-04

**Authors:** Zhong-Yuan Wan, Fang Song, Zhen Sun, Yu-Fei Chen, Wei-Lin Zhang, Dino Samartzis, Chi-Jiao Ma, Lu Che, Xu Liu, M-Azam Ali, Hai-Qiang Wang, Zhuo-Jing Luo

**Affiliations:** Department of Orthopaedics, Xijing Hospital, Fourth Military Medical University, Xi’an, P. R. China; Department of Stomatology, The General Hospital of the Second Artillery Corps of Chinese PLA, Beijing, P. R. China; Department of Orthopaedics, The General Hospital of Air Force of PLA, Beijing, P. R. China; Department of Orthopaedics and Traumatology, The University of Hong Kong, Pokfulam, Hong Kong, SAR China; Aerospace Medical School, Fourth Military Medical University, Xi’an, P. R. China; Bioengineering and Clothing and Textile Sciences, Department of Applied Sciences, University of Otago, Dunedin, New Zealand

## Abstract

**Introduction:**

In addition to the well-known short noncoding RNAs such as microRNAs (miRNAs), increasing evidence suggests that long noncoding RNAs (lncRNAs) act as key regulators in a wide aspect of biologic processes. Dysregulated expression of lncRNAs has been demonstrated being implicated in a variety of human diseases. However, little is known regarding the role of lncRNAs with regards to intervertebral disc degeneration (IDD). In the present study we aimed to determine whether lncRNAs are differentially expressed in IDD.

**Methods:**

An lncRNA-mRNA microarray analysis of human nucleus pulposus (NP) was employed. Bioinformatics prediction was also applied to delineate the functional roles of the differentially expressed lncRNAs. Several lncRNAs and mRNAs were chosen for quantitative real-time PCR (qRT-PCR) validation.

**Results:**

Microarray data profiling indicated that 116 lncRNAs (67 up and 49 down) and 260 mRNAs were highly differentially expressed with an absolute fold change greater than ten. Moreover, 1,052 lncRNAs and 1,314 mRNAs were differentially expressed in the same direction in at least four of the five degenerative samples with fold change greater than two. Kyoto Encyclopedia of Genes and Genomes (KEGG) pathway analysis for the differentially expressed mRNAs indicated a number of pathways, such as extracellular matrix (ECM)-receptor interaction. A coding-noncoding gene co-expression (CNC) network was constructed for the ten most significantly changed lncRNAs. Annotation terms of the coexpressed mRNAs were related to several known degenerative alterations, such as chondrocyte differentiation. Moreover, lncRNAs belonging to a particular subgroup were identified. Functional annotation for the corresponding nearby coding genes showed that these lncRNAs were mainly associated with cell migration and phosphorylation. Interestingly, we found that Fas-associated protein factor-1 (*FAF1*), which potentiates the Fas-mediated apoptosis and its nearby enhancer-like lncRNA RP11-296A18.3, were highly expressed in the degenerative discs. Subsequent qRT-PCR results confirmed the changes.

**Conclusions:**

This is the first study to demonstrate that aberrantly expressed lncRNAs play a role in the development of IDD. Our study noted that up-regulated RP11-296A18.3 highly likely induced the over-expression of *FAF1*, which eventually promoted the aberrant apoptosis of disc cells. Such findings further broaden the understanding of the etiology of IDD.

**Electronic supplementary material:**

The online version of this article (doi:10.1186/s13075-014-0465-5) contains supplementary material, which is available to authorized users.

## Introduction

Low back pain is a global burden, affecting the quality of life and resulting in serious socioeconomic consequences [1,2]. The main cause of low back pain is intervertebral disc degeneration (IDD) (Online Mendelian Inheritance in Man(OMIM) # 603932) [3]. Numerous studies indicate that a variety of cellular events are disturbed in the progression of IDD, ranging from matrix synthesis to cytokine expression [4]. Underlying these alterations there is the dysregulated gene expression of particular molecules. Large-scale gene expression studies have unveiled that a great number of coding genes are differentially expressed in IDD, parts of which were demonstrated as playing significant roles in IDD [5,6]. With the progress of genetic and proteomic tools, our understanding of gene dysregulation in IDD has been expanded greatly. Several treatment strategies targeting dysregulated genes have been proposed in IDD animal models with encouraging results [7,8]. However, dysregulation of gene expression is a very complex process. Previous studies suggest that alterations of several regulatory factors at different levels result in the aforementioned eventual gene dysregulation [9]. Among these factors, aberrantly expressed regulatory noncoding RNAs have attracted considerable attention in recent years.

Noncoding RNAs are a diverse group of transcriptional outputs of the mammalian genome without protein-coding function. A growing body of evidence suggests that noncoding RNAs participate in a multitude of processes coordinating gene expression [10]. There are two major groups of noncoding RNAs, short noncoding RNA, which has less than 200 nucleotides, and large noncoding RNA (lncRNA), which contain over 200 nucleotides, some more over 100,000 nucleotides [11]. miRNAs, the well-understood short noncoding RNAs, function by binding to the 3'-untranslated region of their target mRNAs, triggering either translation inhibition or mRNA degradation [12]. miRNAs have different expressions in numerous diseases [13,14]. In fact, miRNAs have been noted to play a role in IDD development. We have previously addressed the expression profiles of miRNAs in IDD [15].

In the past decade, growing numbers of lncRNAs have been recognized and shown to function in virtually every aspect of human biology [16], including chromosomal dosage compensation, control of imprinting, chromatin modification, chromatin structure, transcription, splicing, translation, cellular differentiation, integrity of cellular structures, cell cycle regulation, intracellular trafficking, reprogramming of stem cells and heat shock response [17]. Multiple lines of evidence also strongly link mutation and dysregulation of lncRNAs to a range of human diseases, including neurodegeneration and cancer [18]. However, to date, there have been no studies addressing the expression profiles of lncRNAs in IDD. As such, in this study, we performed a lncRNA-mRNA microarray analysis to identify the expression profiles of lncRNAs in the nucleus pulposus (NP) in IDD. A series of bioinformatics prediction methods were applied for the function annotation of the differentially expressed lncRNAs.

## Methods

### Sample collection

The study procedures were approved by the Ethics Review Board of Xijing Hospital (number 20111103-7) and the study has been carried out in accordance with the Declaration of Helsinki (2008) of the World Medical Association. Degenerative NP samples were derived from patients with IDD. Patients with IDD combined with degenerative spinal stenosis, idiopathic scoliosis, tumors, infections or previous lumbar disc surgery were excluded from this study. Non-degenerative specimens were obtained from cadaveric donors. All patients or relatives of the cadaveric donors were fully informed of the aim and protocols of the study, and agreed to participate in the study. Subsequently, written informed consent was obtained. The degenerative condition of IDD was evaluated on magnetic resonance imaging (MRI) (for the cadavers MRI data were collected from records) according to the Pfirrmann’s grading system [19]. All specimens were obtained from the lumbar spine (Table 1). Samples obtained from graded as 1 from cadaveric donors were classified as the normal group, samples graded as 4 or 5 from patients were included in the IDD group. All the specimens were collected within 3 hours after disc excision (for discs from patients with IDD) or death (for discs from cadaveric donors), following rinsing with phosphate-buffered saline, the NP tissue was carefully collected under a stereoscopic microscope. Five degenerative and five normal samples were obtained from five patients and five cadaveric donors respectively.

**Table 1:** **Study-related specimen information**

**Sample number**	**Disc level**	**Pfirrmann grade**	**Gender**	**Age**
Deg -1	L4 to L5	5	F	32
Deg-2	L3 to L4	5	M	38
Deg-3	L3 to L4	4	M	42
Deg-4	L5 to S1	5	M	45
Deg-5	L4 to L5	4	F	27
Nor -1	L3 to L4	1	M	33
Nor-2	L3 to L4	1	M	35
Nor-3	L4 to L5	1	M	41
Nor-4	L5 to S1	1	F	43
Nor-5	L4 to L5	1	M	52

Deg, degenerative sample; Nor, normal sample. L, lumbar; S, sacral.

### RNA isolation and quality control assay

Total RNA was extracted from homogenized samples with TRIzol® reagent (Invitrogen Life Technologies, San Diego, CA, USA) according to the manufacturer’s protocol. Then the RNA was cleaned with a RNasey Mini Kit (Qiagen, Germantown, MD, USA). RNA quantity was measured using the NanoDrop ND-1000 spectrophotometer (NanoDrop Technologies, Wilmington, DE, USA). RNA integrity and gDNA contamination were assessed by denaturing agarose gel electrophoresis.

### Microarray analysis

The microarray used in the current study was Arraystar Human LncRNA Array v2.0 (Arraystar, Rockville, MD, USA), which contains 33045 lncRNAs and 30215 protein-coding transcripts. The lncRNAs were collected from several authoritative data sources, including RefSeq, UCSC Known genes, Ensembl and related literature. The mRNAs were obtained from RefSeq (March 2011). Each transcript is represented by a specific exon or splice junction probe which can identify individual transcripts accurately. Positive probes for housekeeping genes and negative probes were also printed onto the array for hybridization quality control.

The microarray analysis was performed according to the standard procedure. Briefly, mRNA was purified from total RNA after removal of rRNA using mRNA-ONLY™ Eukaryotic mRNA Isolation Kit (Epicentre, Madison, WI, USA). Subsequently, each sample was amplified and transcribed into fluorescent cRNA along the entire length of the transcripts without 3’ bias, utilizing a random priming method. The labeled cRNAs were purified by RNeasy Mini Kit (Qiagen). The concentration and specific activity of the labeled cRNAs (pmol Cy3/μg cRNA) were measured by NanoDrop ND-1000. One μg of each labeled cRNA was fragmented by adding 5 μl 10 × blocking agent and 1 μl of 25 × fragmentation buffer, then the mixture was heated at 60°C for 30 minutes; finally, 25 μl 2 × GE hybridization buffer was added to dilute the labeled cRNA. Fifty μl of hybridization solution was dispensed into the gasket slide and assembled to the lncRNA expression microarray slide. The slides were incubated for 17 hours at 65°C in an Agilent hybridization oven (Agilent Technologies, Santa Clara, CA, USA). The hybridized arrays were washed, fixed and scanned with the Agilent DNA Microarray Scanner (Agilent Technologies).

### Data analysis

Agilent Feature Extraction software (version 11.0.1.1) (Agilent Technologies) was used to analyze acquired array images. Quantile normalization and subsequent data processing were performed with the Gene Spring GX v11.5.1 software package (Agilent Technologies). After quantile normalization of the raw data, lncRNAs and mRNAs that at least 5 out of 10 samples have flags in Present or Marginal (All Targets Value) were chosen for further data analysis. The normalized intensity for each lncRNA and mRNA was presented as a log2-transformed pattern. The microarray data have been deposited in the National Center for Biotechnology Information Gene Expression Omnibus (GEO) [GSE56081: GEO]. Differentially expressed lncRNAs and mRNAs between two groups were identified through fold-change filtering and Student’s *t*-test. Multiple testing correction was performed by calculating the Benjamini-Hochberg false discovery rate (FDR). Fold-change greater than two, a *P*-value <0.05 (two-tailed) and an FDR <0.05 were considered the criteria for differential expression. A positive fold-change value indicates upregulation and a negative fold-change value indicates downregulation.

### lncRNA classification and subgroup analysis

The lncRNAs were classified into different subgroups and analyzed according to their various interaction mechanisms with mRNAs. Human homeobox transcription factors (HOX) cluster mRNAs and lncRNAs were screened based on the previous study [20]. lncRNAs with enhancer-like function were identified using GENCODE annotation [21] of the human genes [22]. The consideration of selection of lncRNAs with enhancer-like function excluded transcripts mapping to the exons and introns of annotated protein coding genes, the natural antisense transcripts, overlapping the protein coding genes and all known transcripts. The large intervening noncoding RNAs (lincRNAs) were also identified according to John Rinn's papers [23,24].

### Functional annotation of the differentially expressed mRNAs

Kyoto Encyclopedia of Genes and Genomes (KEGG) enrichment analysis was used to analyze the biological pathways, involving the differentially expressed mRNAs. The Database for Annotation, Visualization and Integrated Discovery (DAVID) (version 6.7) was applied to investigate the functional enrichment condition of the differentially expressed mRNAs, which were upregulated or downregulated shared by four of the five degenerative samples or in all degenerative samples with the normalized intensities altered more than 2-fold compared with the normal samples. We also used the DAVID for functional annotation of the nearby coding genes of the differentially expressed Rinn’s lincRNAs and enhancer-like lncRNAs. The default annotation category and the high-classification stringency settings were selected. Functional annotation clusters with an enrichment score >1.3 were considered significant [25].

### Quantitative real-time PCR (qRT-PCR) assay

Although microarray is an excellent tool for target-gene screening discovery, its credibility is limited by the inconsistency of the technique, weak sensitivity and specificity. Thus, candidate target genes are required to be validated with highly reliable biotechniques, such as qRT-PCR [26].

Four randomly selected differentially expressed lncRNAs lincRNA-SLC20A1-1 [lincRNA:BI522654], RP11-296A18.3 [Ensembl:ENST00000366181], KB-1683C8.1 [Ensembl:ENST00000492365], lincRNA-BTG2 [lincRNA:X75546] and two differentially expressed mRNAs *CASP8* [RefSeq_coding:NM_001080124], *FAF1* [RefSeq_coding:NM_007051] were chosen to validate the gene chip results using qRT-PCR. The lncRNAs, mRNAs and specific primers are noted in Table 2. The qRT-PCR analysis was performed according to the following procedure. Briefly, total RNA was extracted from NP tissue using TRIzol® reagent (Invitrogen). Then cDNAs were synthesized from total RNA using Super Script™ III Reverse Transcriptase (Invitrogen) on Gene Amp® PCR System 9700 (Applied Biosystems, Foster City, CA, USA). qRT-PCR was performed on a ViiA™ 7 Real-time PCR System (Applied Biosystems) with a total reaction volume of 10 μl, including 5 μl 2X PCR master mix (Superarray Bioscience Corporation, Frederick, MD, USA), 0.5 μl of PCR forward primer (10 μM), 0.5 μl of PCR reverse primer (10 μM), 2 μl of cDNA and 2 μl of double-distilled water. The thermal cycling conditions were as follows: initial denaturation at 95°C for 10 minutes, followed by 40 cycles of 10 seconds of denaturing at 95°C, 60 seconds of annealing at 60°C and 15 seconds of extension at 72°C. Each qRT-PCR reaction was done with a technical triplicate. The expression levels of the target lncRNAs and mRNAs were normalized to that of glyceraldehyde-3-phosphate dehydrogenase (*GAPDH*) in cDNA samples.

**Table 2:** **lncRNAs and mRNAs for qRT-PCR validation and the primer sequences**

**Gene**	**Sense**	**Sequence 5′ → 3’**
lincRNA-SLC20A1-1	F	CATCCTCCCACCCAATCATC
	R	GGACCTCCAGCAAACACCAG
RP11-296A18.3	F	GTTCAAGTGCTTCTCCTG
	R	GCTTATGCCTGTAATCCC
KB-1683C8.1	F	GGCCAAGGACAAAGAATGGA
	R	CATGGGCAGGGAGAAGAGATAG
lincRNA-BTG2	F	CCACTACCTCCGCAGCCA
	R	GCCCTTCATCCACCCCATA
CASP8	F	TTCAGAAGAAGGAGCAG
	R	CTGTCCAGTTGTTCC
FAF1	F	AGAGCAAAGAGGGAAG
	R	AAGAACTCGCCACTG
GAPDH	F	GGGAAACTGTGGCGTGAT
	R	GAGTGGGTGTCGCTGTTGA

### Co-expression network construction

A coding-noncoding gene co-expression (CNC) network was constructed based on the Pearson correlation calculation for the normalized signal intensity of differentially expressed lncRNAs and mRNAs. The top five upregulated (RP11-475I24.4 [Ensembl:ENST00000461676], CTB-28 J9.3 [Ensembl:ENST00000514459], AC005077.14 [Ensembl:ENST00000421546], [misc_RNA:AK023939] and [misc_RNA:G43223]) and top five downregulated lncRNAs (HOTAIR [RefSeq_NR:NR_003716], nc-HOXA13-96 [HOX cluster:nc-HOXA13-96-], AC131180.3 [Ensembl:ENST00000451959], [misc_RNA:AK096112] and nc-HOXD1-48 [HOX cluster:nc-HOXD1-48+]) were selected as the sources of the network. The significant correlated mRNAs were chosen as the targets to build the network with an absolute value of the Pearson correlation coefficient >0.99 using the program Cytoscape (version 3.0.1). For coding genes with several transcripts, the median value of different transcripts was taken as the value of the genes expression. Yellow round rectangle nodes represent the upregulated lncRNAs in the network, while the blue nodes represent the downregulated lncRNAs. Pink round nodes represent the upregulated mRNAs, while the green round nodes represent the downregulated mRNAs. Solid lines indicate positive correlation, whereas dashed lines indicate negative correlation. Correlation degrees for each pair of nodes were weighted and delineated by the length of the lines. KEGG enrichment analysis and Gene Ontology (GO) analysis were used to annotate the function roles of mRNAs significantly correlated with the aforementioned lncRNAs.

### Statistical analysis

Statistical analysis was performed using SPSS 17.0 software package. Student’s *t*-test was applied to analyze the expression difference of the particular lncRNAs or mRNAs in microarray and PCR analysis. Moreover, the Benjamini-Hochberg FDR (the FDR cutoff was 0.05) was used for multiple-testing correction. A *P*-value <0.05 (two tailed) was considered statistically significant.

## Results

### Microarray data

**Figure 1:**
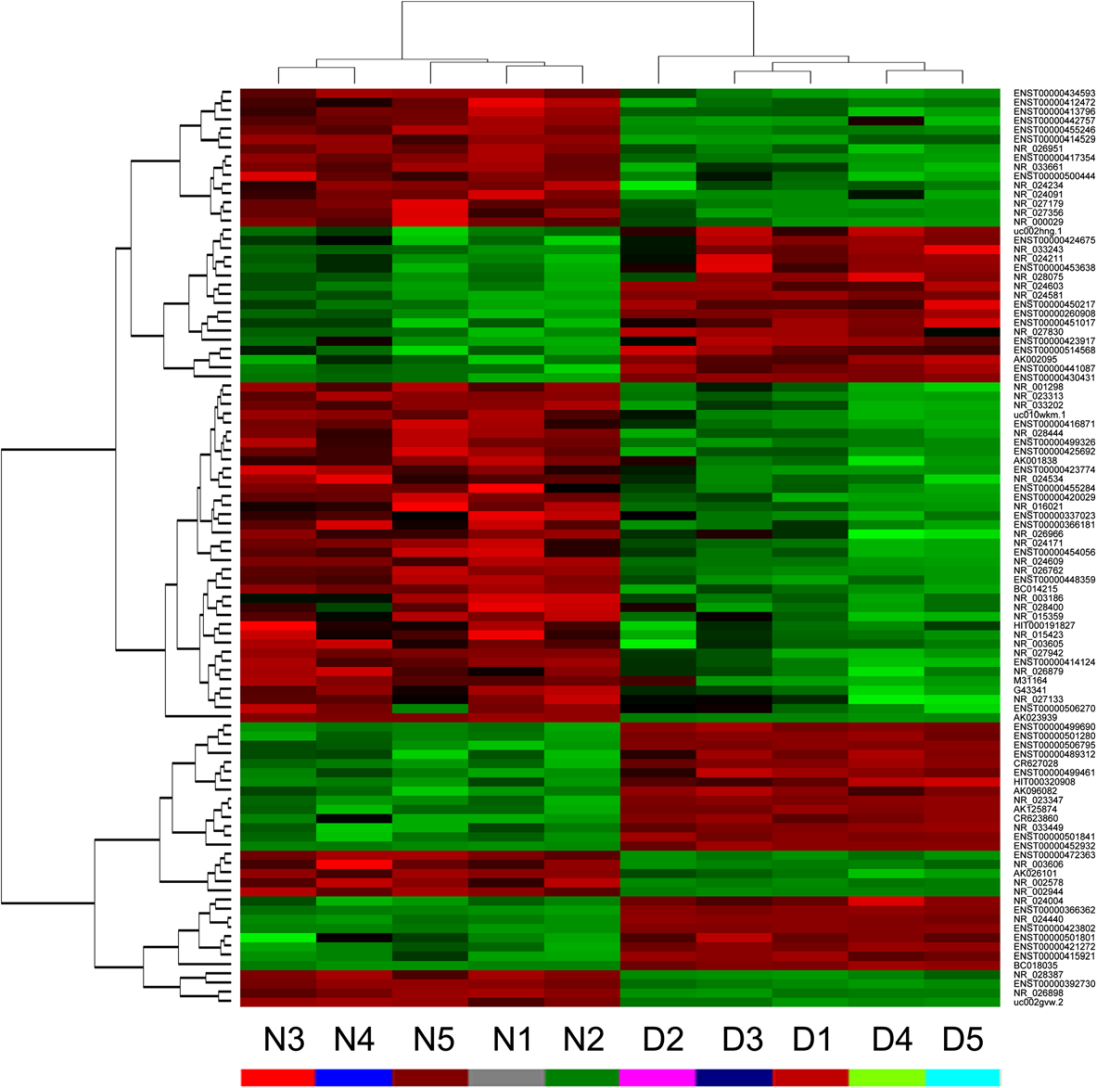
**Hierarchical clustering dendrogram and heat map.** Hierarchical clustering based on lncRNA expression value showed a distinguishable gene expression profiling among samples. Green strip indicates high relative expression and red strip indicates low relative expression. N1 represents normal sample 1, D1 represents degenerative sample 1, and so on.

Results of the quality control assay showed that total RNAs extracted from the samples qualified for subsequent experiments. The hierarchical clustering was performed based on the lncRNA expression values in the microarray (Figure 1). Microarray data indicated that a total of 1,806 lncRNAs and 2,307 mRNAs were significantly differentially expressed in IDD compared with the control group. Among them, 1,357 lncRNAs were upregulated with 449 lncRNAs downregulated and 1,694 mRNAs were upregulated with 613 mRNAs downregulated. The top 10 upregulated and top 10 downregulated lncRNAs are listed in Table 3. In addition, 116 lncRNAs (67 upregulated and 49 downregulated) and 260 mRNAs (137 upregulated and 123 downregulated) were highly differentially expressed with an absolute fold-change >10 (see Additional file 1). Furthermore, 237 lncRNAs were upregulated shared by four of the five degenerative samples with the normalized intensities altered more than 2-fold compared with all the normal specimens (see Additional file 2). On the other hand, 629 lncRNAs were upregulated in all degenerative specimens with a fold-change >2 compared with all the control samples (see Additional file 2). Among the differentially downregulated lncRNAs, 42 lncRNAs were downregulated in four of the five degenerative samples; whereas 144 lncRNAs were downregulated in all degenerative samples (see Additional file 2). There were 323 over-expressed and 75 downregulated mRNAs in four of the five degenerative samples, whereas there were 670 over-expressed mRNAs and 246 downregulated mRNAs in all degenerative samples with a fold change >2 compared with all the normal samples (see Additional file 2).

**Table 3:** **The top 10 upregulated and downregulated lncRNAs detected by microarray analysis in the degenerative group**

**Sequence name**	**Source database**	**Fold-change**	***P*** **-value**
**Upregulated lncRNAs**			
ENST00000461676	Ensembl	135.56903	6.51E-07
ENST00000514459	Ensembl	97.71137	1.78E-05
ENST00000421546	Ensembl	73.82017	1.19E-05
AK023939	misc_RNA	52.0571	6.00E-07
G43223	misc_RNA	38.324024	1.47E-06
ENST00000446763	Ensembl	36.52328	6.72E-04
ENST00000432925	Ensembl	34.249966	1.68E-03
uc003mjv.3	UCSC_knowngene	33.52595	6.32E-05
U94385	RNAdb	31.505856	3.28E-04
chr8:87319334-87331334+	lincRNA	29.106977	6.94E-05
**Downregulated lncRNAs**			
NR_003716	RefSeq_NR	148.53123	7.83E-05
nc-HOXA13-96-	HOX cluster	111.87458	1.68E-06
ENST00000451959	Ensembl	96.609	2.52E-05
AK096112	misc_RNA	95.62264	8.97E-05
nc-HOXD1-48+	HOX cluster	89.8041	1.86E-05
BG897081	lincRNA	82.24899	4.28E-06
uc003tjk.2	UCSC_knowngene	77.483154	2.58E-06
NR_027154	RefSeq_NR	76.309654	3.74E-07
BX646285	lincRNA	74.3615	9.86E-05
AK125976	misc_RNA	72.67137	1.42E-04

### lncRNA classification and subgroups

In 2007, Rinn *et al*. described 407 HOX transcripts in the four HOX loci, including 101 exons, 75 introns and 231 intergenic transcripts [20]. As a family of highly conserved transcription factors, the HOX transcripts were originally discovered to regulate body development. The mutation and dysregulation of HOX transcripts pertain to a number of diseases, ranging from limb malformation to hematologic malignancies [27,28]. In the current study, we detected 160 HOX coding transcripts in all samples, 16 of which were differentially expressed in IDD compared with the control group (fold-change >2, *P* <0.05, FDR <0.05). Additionally, 292 noncoding transcripts were detected with 29 differentially expressed transcripts in IDD (fold-change >2, *P* <0.05, FDR <0.05). Both the differentially expressed coding and noncoding transcripts in degenerative samples are shown in Additional file 3.

Ørom and coworkers identified a set of lncRNAs characterized with enhancer-like function, which are expressed in multiple cell lines [22]. By filtering the array data, we detected 951 enhancer-like lncRNAs in all specimens. We noted that 76 lncRNAs were differentially expressed in IDD compared with control (fold-change >2, *P* <0.05, FDR <0.05). Moreover, there were 25 differentially expressed lncRNAs with differentially expressed nearby coding genes (distance <300 kb) (see Additional file 3). In these 25 pairs of differentially expressed lncRNAs and nearby coding genes, 18 pairs were dysregulated in a same direction (up or down).

Thousands of lincRNAs are encoded in the mammalian genome with highly conserved features across mammals [23,24]. These lincRNAs are involved in diverse biological processes, including cell-cycle regulation, immune surveillance and embryonic stem cell pluripotency. Profiling data based on Rinn’s lincRNAs indicated that 190 lincRNAs were differentially expressed in IDD within 2,043 detected lincRNAs (fold-change >2, *P* <0.05, FDR <0.05) (see Additional file 3). It is noteworthy that 63 differentially expressed lincRNAs had differentially expressed nearby coding genes (distance <300 kb) (see Additional file 3).

### Functional annotation results of the differentially expressed mRNAs

**Figure 2:**
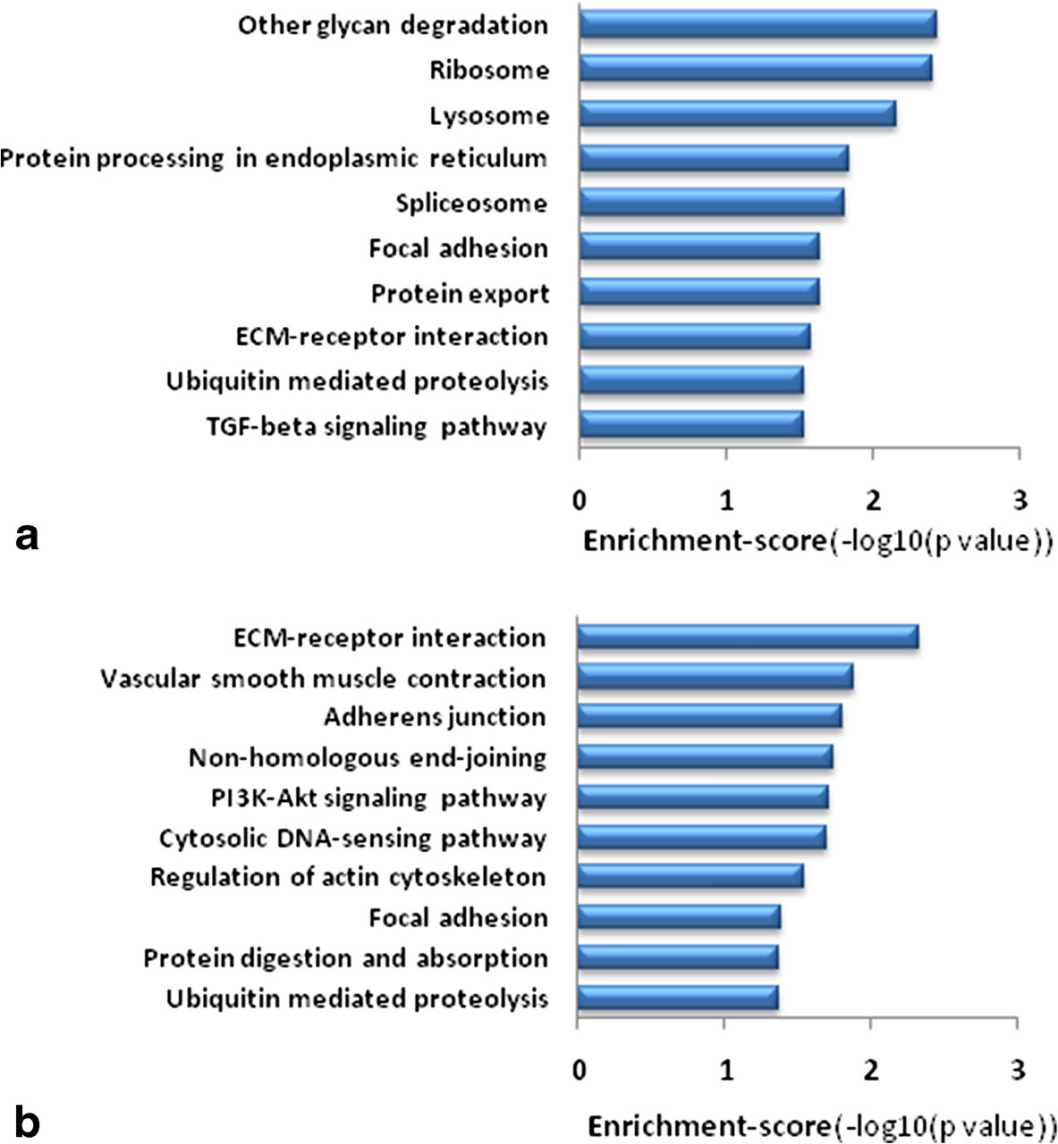
**Results of the KEGG pathway analysis for the differentially expressed mRNAs.** **(a)** The 10 most significant KEGG pathways of the upregulated mRNAs. **(b)** The 10 significant KEGG pathways of the downregulated mRNAs. The length of the column in the figure represents the enrichment score of the each pathway. ECM, extracellular matrix; TGF, transforming growth factor.

Based on the latest version of the KEGG database, we employed KEGG pathway analysis for differentially expressed mRNAs (separately for upregulated mRNAs and downregulated mRNAs). Accordingly, we revealed the biological pathways with significant enrichment of differentially expressed mRNAs. Each *P*-value denoted the significance of the corresponding pathway. The lower the *P*-value, the more significant the pathway was (the *P*-value cutoff was 0.05). Enrichment-score values were also calculated, representing the enrichment importance of pathway IDs. The values equaled -log10 (*P*-values). The greater the enrichment score, the more significant the pathway was. The results of the KEGG pathway analysis were shown in Figure 2 and Additional file 4.

The DAVID functional annotation clustering was applied to investigate the functional enrichment of upregulated or downregulated mRNAs altered more than 2-fold in at least four of the five degenerative samples. The most significant functional clusters are listed in Table 4 and Additional file 4. Table 5 and Additional file 4 summarize the results of DAVID functional annotation clustering for the neighboring coding genes of differentially expressed Rinn lincRNAs and enhancer-like lncRNAs. Enrichment-score values indicated the enrichment importance of clusters. The greater the enrichment score, the more significant the cluster was.

**Table 4:** **DAVID functional annotation clusters of upregulated or downregulated mRNAs in at least four of five degenerative specimens**

**Annotation clusters**	**Cluster-related terms**	**Enrichment score**
**Most significant functional clusters for the mRNAs upregulated in at least four of the five degenerative samples**		
1	Nucleosome core, protein-DNA complex, chromatin assembly, et cetera	3.91
2	Glycosaminoglycan binding, polysaccharide binding, pattern binding, et cetera	3.89
3	Ribosome, translational elongation, cytosolic ribosome	3.63
4	Histone H2A	2.78
5	Basic-leucine zipper domain, DNA-binding region: basic motif	2.58
**Most significant functional clusters for the mRNAs downregulated in at least four of the five degenerative samples**		
1	Negative regulation of gene expression, negative regulation of transcription, negative regulation of nucleobase, nucleoside, nucleotide and nucleic acid metabolic process, et cetera	1.60
2	Cytosolic ribosome, ribosome, ribosomal subunit, et cetera	1.51
3	Phosphoglycerate mutase, Tele-phosphohistidine intermediate, Phosphoglycerate mutase, et cetera	1.49
4	Purine nucleotide biosynthetic process, purine nucleotide metabolic process, nucleotide biosynthetic process, et cetera	1.44
5	Proteasomal ubiquitin-dependent protein catabolic process, proteasomal protein catabolic process, ubiquitin-dependent protein catabolic process, et cetera	1.40

**Table 5:** **DAVID functional annotation clusters of nearby coding genes of differentially expressed Rinn's lincRNAs and enhancer-like lncRNAs**

**Annotation clusters**	**Cluster-related terms**	**Enrichment score**
1	Phosphorylation, phosphorus metabolic process, phosphate metabolic process	1.79
2	Regulation of phosphorylation, regulation of phosphorus metabolic process, regulation of phosphate metabolic process	1.76
3	Cellular respiration, mitochondrial ATP synthesis coupled electron transport, ATP synthesis coupled electron transport, et cetera	1.59
4	Regulation of cell migration, regulation of locomotion, regulation of cell motion	1.33

### qRT-PCR validation

**Figure 3:**
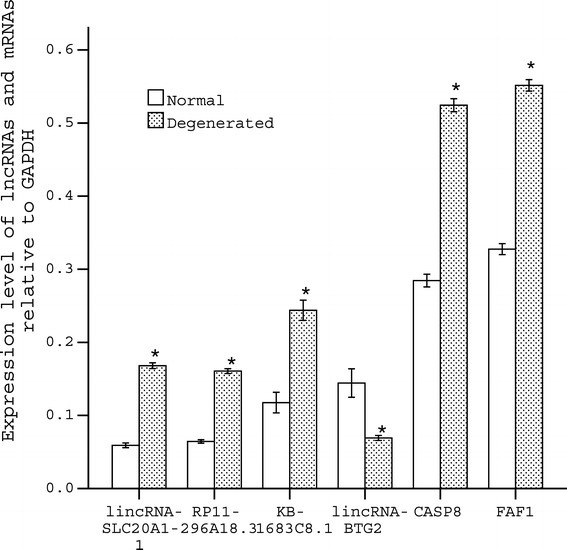
**QRT-PCR confirmation results of the four lncRNAs and two mRNAs.** The four lncRNAs lincRNA-SLC20A1-1, RP11-296A18.3, KB-1683C8.1, lincRNA-BTG2 and two mRNAs *CASP8*, *FAF1* were chosen for the validation of the gene chip results using qRT-PCR. The height of the columns in the figure represents the lncRNA expression level relative to glyceraldehyde-3-phosphate dehydrogenase; the bars represent standard deviation. Normal represents the normal disc group; Degenerated represents the degenerated disc group.*P <0.05 versus the normal group.

To validate the microarray results, four lncRNAs and two mRNAs were selected for the qRT-PCR analysis in 12 human lumbar disc NP samples (6 degenerative and 6 normal samples: detailed sample information is not provided in the paper). The expression of lincRNA-SLC20A1-1 (*P* <0.05), RP11-296A18.3 (*P* <0.05), KB-1683C8.1 (*P* <0.05), *CASP8* (*P* <0.05) and *FAF1* (*P* <0.05) were significantly increased in degenerative discs compared with the normal discs, whereas the expression of lincRNA-BTG2 (*P* <0.05) was significantly decreased (Figure 3). The fold-changes of the normalized intensities were 5.99, 3.21, 2.82, 0.168, 2.42 and 2.17, respectively, for the four lncRNAs (lincRNA-SLC20A1-1, RP11-296A18.3, KB-1683C8.1 and lincRNA-BTG2) and two mRNAs (*CASP8* and *FAF1*) in the gene chip analysis between the IDD and the normal group. For the expression levels in qRT-PCR analysis, the fold-changes were 2.83, 2.50, 2.08, 0.46, 1.84 and 1.68 respectively. The data for qRT-PCR were consistent with the microarray results.

### CNC network

A CNC network was built for the ten differentially expressed lncRNAs and hundreds of differentially expressed mRNAs based on the degree of correlation (see Additional file 5). There were 197 nodes in the network, comprising 10 lncRNAs nodes and 187 mRNAs nodes. These 197 RNAs combined into 673 pairs of co-expressed lncRNAs and mRNAs (see Additional file 6).

KEGG biological pathway analysis indicated that 187 mRNAs were mainly pertinent to the pathways of ribosome and antigen processing and presentation (see Additional file 6). GO terms with regard to these mRNAs are also listed in Additional file 6. Importantly, GO terms of remarkable research interest were found in the list, encompassing translational elongation, negative regulation of cell differentiation, cartilage development, negative regulation of signal transduction, chondrocyte differentiation, collagen fibril organization, posttranscriptional regulation of gene expression, regulation of apoptosis and proteoglycan metabolic process. One CNC network was constructed for each of the aforementioned 10 lncRNAs (Additional file 7).

## Discussion

In the current study, by means of microarray analysis and in-depth data profiling we found that a large amount of lncRNAs and mRNAs were differentially expressed in degenerative discs. Succeeding qRT-PCR confirmation results of the four randomly selected lncRNAs matched well with the microarray data, demonstrating high credibility for the lncRNAs microarray analysis.

Similar to our findings, previous microarray studies on gene expression in degenerative discs reveal that many mRNAs are aberrantly expressed in the degeneration process [6]. Functional annotation for these differentially expressed mRNAs may provide a precise perspective to comprehend the biological pathways and cellular events of the mRNAs involved in IDD. Based on such a consideration, several bioinformatics methods such as KEGG pathway analysis were employed in our study. KEGG pathway analysis showed that not only the upregulated mRNAs were involved in the biological pathways, including focal adhesion, extracellular matrix (ECM)-receptor interaction, adherens junction and ubiquitin-mediated proteolysis, but the downregulated mRNAs also participated in the four pathways. This indicates that biological processes and the consequence of the activity of the aforementioned four pathways in IDD are significantly different from those in normal discs. In addition pathway terms, such as other glycan degradation, antigen processing and presentation, and transforming growth factor-beta signaling pathways, were observed in the results of the KEGG pathways analysis for the upregulated mRNAs. Breakdown of the immune-privilege status of the discs has been noted as an essential pathogenesis factor contributing to the IDD [29]. Once the components of discs are exposed to the immune system, such materials will be recognized as foreign bodies and processed by the antigen presenting cells (APCs) and presented to the T lymphocytes, inducing autoimmune attack on the disc. Increased expression of several genes, for example, *CD74* and *KIR2DS2* were observed in the pathway of antigen processing and presentation. CD74 plays a critical role in antigen presentation from APCs to CD4-positive T lymphocytes by influencing the expression and antigen peptide loading of the major histocompatability complex (MHC) class II molecules on APCs [30]. Moreover, subsequent studies indicate that CD74 is involved in the release of inflammatory cytokines induced by macrophage migration inhibitory factor and associated with the Modic changes of cartilage endplate degeneration in IDD [31]. KIR2DS2 plays a supporting role in natural killer cell-mediated cytotoxicity. Meanwhile, the increased expression of KIR2DS2 can reduce the threshold of T-cell activation and result in an increased risk of rheumatoid arthritis [32].

Further data analyses showed in total about half of the mRNAs (1,314 mRNAs) were upregulated (993, with 323 in four of the five degenerative samples, and 670 in all degenerative samples) or downregulated (321, with 75 in four of the five degenerative samples, and 246 in all degenerative samples) in at least four of the five degenerative samples with normalized intensities, altering more than 2-fold compared with the non-degenerative samples. DAVID functional annotation clustering was employed for the functional annotation of the mRNAs. Among the clusters, terms of membrane-bound vesicles were also noted, which pertain to upregulated genes in IDD development [33]. Interestingly, several other clusters were observed in our study, including apoptosis and negative regulation of gene expression. These pathologic findings are consistent with previous studies in IDD [34,35].

In addition to the numerous differentially expressed mRNAs, thousands of lncRNAs were detected and differentially expressed in the degenerative samples. The variation of lncRNA expression in the degenerative intervertebral discs implicates that lncRNAs may play a crucial role in the onset and development of IDD. Further analysis revealed that 866 (237 in four of the five degenerative samples, 629 in all degenerative samples) and 186 lncRNAs (42 in four of the five degenerative samples, 144 in all degenerative samples) were upregulated and downregulated in at least four of the five degenerative samples, respectively, with normalized intensities altered more than 2-fold compared with the normal samples. These portions of lncRNAs were highly likely relevant to the occurence and progression of IDD. Moreover, these lncRNAs account for 64% and 41% of the upregulated and downregulated lncRNAs, respectively.

A CNC network was constructed based on the Pearson correlation analysis data of ten differentially expressed lncRNAs and hundreds of differentially expressed mRNAs. The ten lncRNAs comprised the top five upregulated and top five downregulated lncRNAs. GO analysis for the co-expressed mRNAs indicates that lncRNAs play important roles in several pathological alterations of IDD, including matrix remodeling and NP cell apoptosis. Details of the roles of lncRNAs and of how lncRNAs function deserve further study.

Certain lncRNAs with particular features can be divided into several subgroups, such as Rinn lincRNAs and enhancer-like lncRNAs. It has been shown that the transcription of these lncRNAs can affect the expression of their nearby coding genes [22]. Dysregulated lncRNAs are closely linked with a variety of diseases [36,37]. The DAVID functional annotation clustering indicates that these lncRNAs are closely related to various biological processes, such as cell migration and phosphorylation.

As induced by Fas and Fas ligand (FasL) interaction, apoptosis of disc cells plays an important role in IDD [38,39]. In accordance with previously reported upregulated Fas and FasL [40,41], our study indicates that *FAF1* was highly expressed in degenerative discs. Subsequent qRT-PCR results confirmed the expression changes. FAF1 is a multi-domain protein as a member of the Fas death-inducing signaling complex [42]. Over-expression of FAF1 can significantly potentiate Fas-mediated apoptosis and inhibit the degradation of ubiquitinated proteins, resulting in increased cell death [43,44]. Corresponding to the upregulated *FAF1*, we also found that the nearby enhancer-like lncRNA, RP11-296A18.3, was significantly increased in degenerative discs compared with the normal discs. Enhancer-like lncRNAs can activate the proximal promoter and stimulate transcription of their nearby coding genes. Depletion of enhancer-like lncRNAs can repress their neighboring protein-coding genes [22]. Whether upregulated RP11-296A18.3 causes the over-expression of its nearby gene *FAF1* and is eventually involved in the aberrant apoptosis of NP cells in degeneration represents an interesting issue that deserves to be further explored.

There are several limitations in the current study. For one, the scarce number of samples included in the microarray analysis may limit the validity of the array results. Moreover, limited by the research approaches, we merely predicted the function of the differentially expressed lncRNAs, but could not confirm and illustrate the detailed functional roles of these lncRNAs. Function of the identified lncRNAs can be investigated by over-expression and RNA interference approaches *in vitro* and *in vivo* [45]. Selectively upregulating or downregulating the certain lncRNA expression in cell cultures and in living organisms will result in various phenotype and gene expression changes. Depending on these changes, the rough functional roles of lncRNAs can be deduced. Subsequently, detailed function and mechanism of the lncRNAs can be explored by means of approaches such as fluorescence *in situ* hybridization, RNA immunoprecipitation and RIP-Chip, et cetera [26].

## Conclusions

To our knowledge, this is the first study addressing lncRNA expression profiles in IDD. Our findings shed a novel light on our understandings of the pathogenesis of IDD. Further studies should focus on the function and pathogenic mechanisms of the lncRNAs involved in IDD. Advances in the knowledge of lncRNAs in IDD may be greatly valuable for bench to bedside translational work to benefit patients suffering from symptomatic IDD.

## Additional files

Additional file 1
**Highly differentially expressed lncRNAs and mRNAs in microarray analysis.** The files present the lncRNAs and mRNAs differentially expressed with an absolute fold-change >10. Additional file 2
**LncRNAs and mRNAs altered in the same direction in at least four of the five degenerative samples with fold-change >2, respectively.** LncRNAs and mRNAs shared by four of five or in all degenerative specimens with a normalized intensity altered more than 2-fold compared with all the normal samples are shown in the file. Additional file 3
**LncRNAs belonging to particular subgroup.** HOX mRNAs and lncRNAs, enhancer-like lncRNAs and nearby coding genes (distance <300 kb), Rinn's lincRNAs and nearby coding genes (distance <300 kb) are identified and presented. Additional file 4
**Functional annotation results of the differentially expressed mRNAs.** The results of the KEGG pathway analysis for the upregulated and downregulated mRNAs in the degenerative group are shown in this file. DAVID functional annotation clustering analysis results for the mRNAs altered in the same direction in at least four of the five degenerative samples with fold-change >2, respectively, and the nearby coding genes (distance <300 kb) of differentially expressed Rinn's lincRNAs and enhancer-like lncRNAs are also presented in this file. Additional file 5
**CNC network of the ten most significantly changed lncRNAs.** A CNC network was constructed for the ten most significantly changed lncRNAs. The network includes 197 nodes; 10 nodes were lncRNAs, the other 187 were mRNAs. These 202 nodes combined into 673 pairs of co-expressed lncRNAs and mRNAs. Additional file 6
**CNC network information and the KEGG biological pathway and GO analysis results of the co-expressed mRNAs.** LncRNAs and mRNAs of the CNC network constructed for the ten most significantly changed lncRNAs, co-expressed lncRNAs and mRNAs pairs and the KEGG biological pathway and GO analysis results of the 187 co-expressed mRNAs are presented. Additional file 7
**CNC networks of the ten most significantly changed lncRNAs.** One CNC network was constructed for each of the ten most significantly changed lncRNAs and these are shown in this file.

## Abbreviations

APC: antigen presenting cell
CNC: coding-noncoding gene co-expression
DAVID: Database for Annotation Visualization and Integrated Discovery
ECM: extracellular matrix
FAF1: Fas-associated protein factor-1
FasL: Fas ligand
FDR: false discovery rate
GAPDH: glyceraldehyde-3-phosphate dehydrogenase
GEO: Gene Expression Omnibus
GO: Gene Ontology
HOX: human homeobox transcription factors
IDD: intervertebral disc degeneration
KEGG: Kyoto Encyclopedia of Genes and Genomes
lincRNAs: large intervening noncoding RNAs
lncRNAs: long noncoding RNAs
miRNAs: microRNAs
MRI: magnetic resonance imaging
NP: nucleus pulposus
qRT-PCR: quantitative real-time polymerase chain reaction
