# Supplementary figures and images for: Aberrantly expressed long noncoding RNAs in human intervertebral disc degeneration: a microarray related study
Source: Arthritis Res Ther. 2014 Oct 4;16(5):465. doi: 10.1186/s13075-014-0465-5 (PMC4201740; doi:10.1186/s13075-014-0465-5)

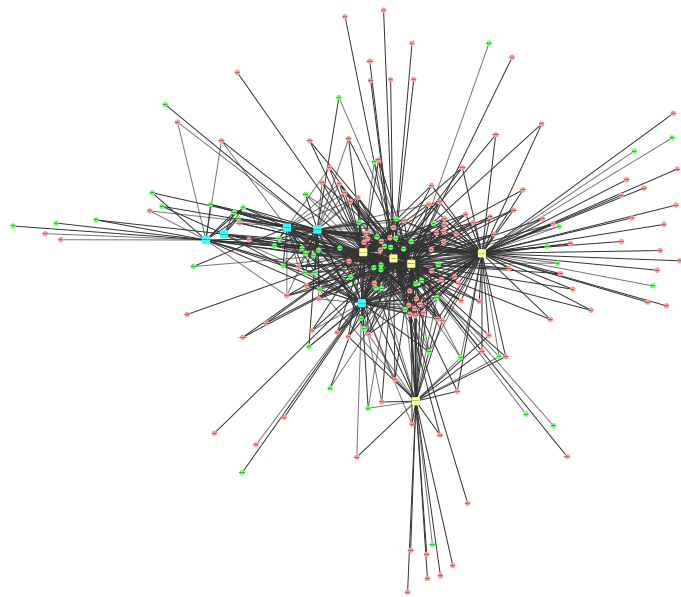

Supplement: Additional file 5 — CNC network of the ten most significantly changed lncRNAs. A CNC network was constructed for the ten most significantly changed lncRNAs. The network includes 197 nodes; 10 nodes were lncRNAs, the other 187 were mRNAs. These 202 nodes combined into 673 pairs of co-expressed lncRNAs and mRNAs. [file 13075_2014_465_MOESM5_ESM.pdf]
